# Concrete language enhances sharing of social media posts on Twitter, Reddit, and experimentally

**DOI:** 10.1177/17470218251392831

**Published:** 2025-10-21

**Authors:** Danyang Hu, Charlie Pilgrim, Weize Zhao, Thomas T. Hills

**Affiliations:** 1 Department of Psychology, University of Warwick, Coventry, UK; 2 Department of Mathematics, University of Leeds, UK

**Keywords:** concreteness, psycholinguistics, information-sharing, upvotes

## Abstract

Concrete language, which has the property of readily evoking a mental image or sensory experience, has been extensively studied in language and is understood to facilitate processing speed, memory, and understanding. Previous research points to a preference for concreteness. Through a comprehensive approach that combines big-data analysis and experimental methods, we investigate the preference for concreteness in posts from two social media platforms—Twitter and Reddit—and in decisions made by participants in a two-alternative forced-choice experiment. In Study 1, we analysed data from 15 million Twitter posts. The overall results show that posts containing words that are more concrete are more likely to be retweeted. In Study 2, we scraped over 50,000 posts from Reddit across different subreddits and found that more concrete posts tend to have more upvotes (i.e., approval). Both studies also showed consistent effects for words acquired later in life and rated as more arousing. The magnitude of these effects varied across topics. Finally, to demonstrate the causal influence of concreteness, Study 3 is a pre-registered, controlled behavioural experiment where we asked participants to indicate which of two social media posts they would be more likely to share, with both posts constructed from a real post to have either higher or lower concreteness than the original. Participants preferred more-concrete statements, and the difference in concreteness between statements positively predicted choices. Our investigation sheds light on cognitive mechanisms underlying online information sharing and is consistent with an information competition theory, in which more easily processed information is preferred.

## Introduction

Contemporary information markets are highly competitive, with numerous individuals and industries vying for consumer attention. This has led to proposals that information competition drives the market for more attention-grabbing information (Davenport & Beck, 2001; Hansen & Haas, 2001; Hills, 2019; Pilgrim et al., 2024). This competition for attention has been conceptualized as a form of “information overload,” where the sheer volume of content creates a noisy environment in which only certain types of messages succeed (Pothos et al., 2021). Here, “noise” refers not merely to literal interference but to the presence of irrelevant or competing content that dilutes attention and reduces the signal-to-noise ratio for any given message (Hills, 2019; Shannon, 1949). As modern information environments grow denser, “competition” refers to the growing number of information producers and the finite attentional resources of receivers (Evans, 2020; Pilgrim et al., 2024; Simon, 1996; Terranova, 2012). This imbalance forces communicators to adapt their messages in ways that lower cognitive load and maximize impact (Falkinger, 2008; Hefti & Lareida, 2021; Picard & McEwen, 2018). For example, it has been found that information with greater processing ease is better able to capture and hold attention (Berger et al., 2023). The increased use of concrete language—language that is specific, imageable, and easily processed is one of the proposed adaptations of communicators (Connell & Lynott 2012; Winkielman & Cacioppo 2001).

As the number of information producers increases, the kinds of messages they communicate need to change in order to successfully capture the attention of information receivers. Early research on the relationship between efficient information transmission and noise showed that as noise increases messages should alter their form to maximize information for the receiver (Shannon, 1949). If we take noise to include all information unrelated to the message of interest—either as intended by the producer or as preferred by the receiver—then modern information markets are rife with noise. Moreover, this noise is likely to be increasing, as there is ample evidence of an accelerating competition for collective attention even in the last decade (Lorenz-Spreen et al., 2019). Savvy information producers should therefore adapt their messages accordingly. Given this simple observation, one might ask how such theories can be extended to help us better understand what kinds of messages are more likely to be successful.

One way to approach this question is to ask what kinds of language are more readily processed, remembered, and reproduced. Experimental studies in this area have found that concrete language has an advantage in studies of perception, memory and speed of recall (Walker & Hulme, 1999). Concreteness refers to how easy it is to imagine or see the referent of a word in one’s mind’s eye (Brysbaert, Warriner, et al., 2014).

It describes the extent to which a concept can be directly experienced through the senses and mentally visualized. Words that refer to tangible, physically perceptible entities are considered concrete, whereas abstract words typically represent ideas or states that require linguistic explanation rather than sensory perception (Brysbaert, Warriner, et al., 2014). For instance, “laughter” is more concrete than “humour,” and “running” is more concrete than “movement.” Although abstract words can convey broader aspects and subtleties of information, concrete messages are more precise and more readily come to mind. To measure concreteness, researchers ask people to rate words with respect to their concreteness using a definition like the one above (Brysbaert, Stevens, et al., 2014; Paivio et al., 1968). People’s ratings tend to be remarkably reliable. Average ratings are also excellent predictors of processing, memory, and speed of recall, three features that are critical to information sharing and reproduction (Hills, 2019).

The effectiveness of concrete language can potentially be explained by dual-coding theory (Paivio et al., 1994), which posits that people process information through both verbal and non-verbal (imagery-based) systems. Concrete language benefits from activating both systems simultaneously, enhancing comprehension and recall. In contrast, abstract language typically activates only the verbal system, which may be less effective in demanding or uncertain environments. Relatedly, concreteness has been shown to improve memory, reduce ambiguity, and increase the perceived truthfulness and psychological proximity of information (Hansen & Wänke, 2010; Sadoski et al., 1997). These properties suggest that concrete messages are particularly well-suited for capturing attention and promoting information sharing in competitive, high-noise settings such as social media. It has been found that concreteness positively affects sustained attention, which was measured by how far down users scroll for articles (Berger et al., 2023). Therefore, concreteness may not just influence how information is processed, but also whether it is retained and retransmitted—key features in the dynamics of online communication.

In line with this, a growing body of research has shown that concreteness enhances not only comprehension and recall but also the likelihood of information being shared. For example, Li et al. (2024) found that concrete words were more likely to survive both short-term retellings and long-term cultural transmission. In experimental studies, participants are more likely to recall concrete messages (Hanley et al., 2013), process them more quickly (Barber et al., 2013), and rate them as more vivid, familiar, and truthful (Hansen & Wänke, 2010). Beyond memory, concreteness appears to enhance the persuasive power of messages, especially under uncertainty. For instance, research on construal-level theory shows that people prefer concrete over abstract descriptions when events feel psychologically near or personally relevant (Snefjella & Kuperman, 2015; Trope & Liberman, 2010). In the context of social media, where messages compete for attention in rapid, low-effort processing environments, such features may give concrete content a significant advantage. Concreteness also interacts with other known drivers of virality—such as novelty, arousal, and belief-consistency (Berger & Milkman, 2012; Goebel et al., 2024)—by reducing cognitive effort and increasing clarity. Prior exposure to content (Vellani et al., 2023), emotional intensity (Berger, 2011), and perceived usefulness (Epstein et al., 2021) all contribute to sharing behaviour, and concrete messages may be more likely to check these boxes. From this perspective, concreteness is not merely a linguistic feature but a cognitive shortcut: one that helps information break through the noise, stick in memory, and spread.

Applying concreteness and information competition to modern information markets, Hills and Adelman (2015) evaluated the statistical properties of 355 billion words of American English over the last two centuries and found a dramatic and nearly monotonic rise in average levels of concreteness. Hills et al. (2016) further developed a model of this rise based on Shannon’s information theory, showing that increasing “noise” (i.e., competition) enhances the need for information that is processed more quickly by its receiver. In more recent work, Li et al. (2024) found that not only were concrete words more likely to survive over the past two centuries, but they are also more likely to be preserved in story retellings, which were measured using a large dataset of more than 12,000 story retellings by online participants.

Linguistic features could also be one of the drivers for success of fake news, with concreteness directly related to the mechanisms by which people share or believe in fake news: Judgement of truth can be affected by the processing fluency of the information, and concrete information can be recognized faster and have an advantage on comprehensibility (Dechêne et al., 2010). Concrete information also tends to be easier to imagine and recall and therefore might leave the impression of having been previously encountered (Doest & Semin, 2005). This feeling of familiarity tends to contribute to the truth advantage of concrete information, a phenomenon known as the illusory truth effect (Dechêne et al., 2010; Fazio et al., 2015). Furthermore, concreteness helps with imaginability, which itself contributes to perceived truth (Koehler, 1991; Sherman et al., 1985).

According to construal level theory (Liberman & Trope, 2008; Trope & Liberman, 2010), there is an association between likelihood of an event and how concrete the construal is. More likely events tend to be represented more concretely and related to direct experience (Borgida & Nisbett, 1977). Research on reality monitoring identified the richness of perceptual, semantic, and contextual details, and the vividness level of the memory as the most important cues by which people judge if a memory is true or imagined (Johnson, 2006; Johnson et al., 1993; Johnson & Raye, 1981). People also tend to think information with more details is true (DePaulo et al., 2003): the description of concrete feelings and the extent of details are used by the police to judge the accuracy of a testimony (Akehurst et al., 1996). Furthermore, vividly described information tends to have more impact on judgement and decision-making: situations with more details tend to be regarded as more representative and more likely to happen compared with situations with fewer details (Borgida & Nisbett, 1977). Notably, it has been found that people are more likely to share information they approve of and concrete false statements are more likely to be rated as true (Hansen & Wänke, 2010).

Beyond concreteness, words acquired early in life are processed more fluently and are more deeply embedded in semantic memory (Brysbaert & Ellis, 2016). Fake news that is phrased in familiar, early-acquired vocabulary may therefore “feel” more true, enhancing its plausibility and shareability, even in the absence of factual accuracy. Also, humorous information tends to attract more attention regardless of the veracity of the information: it was found in an eye-tracking study that humour could direct people’s attention to both fake news and its correction (Kim et al., 2021). Humour has been shown to increase the likelihood of sharing while reducing cognitive scrutiny, and research suggests that humorous content is more likely to be forwarded (Berger & Milkman, 2012). While deliberation makes people less likely to believe or share fake news (Pennycook et al., 2020), humorous elements/funny content can lower people’s resistance to persuasion (Nabi et al., 2007).

Our goal here is to ask whether or not more concrete language influences sharing behaviour in online social media. By analysing posts from Twitter and Reddit with different topics and subreddits, we investigate if more concrete information is more likely to be shared and upvoted. We do this in two ways. First, we examine posts scraped from Twitter and Reddit and compare their concreteness alongside the extent to which of these posts are retweeted or upvoted. Second, we present a pre-registered, controlled experimental study that manipulates the concreteness of posts and then asks people to select which of two posts they would be more likely to share. Besides concreteness, we also explore the roles of other psycholinguistic properties, including age of acquisition, arousal, dominance, humour and valence, which have been found to be useful covariates in past work (Li et al., 2024).

## Study 1

At the time of writing, Twitter (now X) currently has 368 million users (Twitter logistics, 2024). Quantitative research on Twitter has revealed a wide variety of real-world insights. This includes relationships between tweets and hate crime (Müller & Schwartz, 2023), the sharing of anti-vaccine sentiment (Bonnevie et al., 2021), political polarization (Conover et al., 2011), rumour spreading (Zubiaga et al., 2016), and their potential impacts on democracy (Lorenz-Spreen et al., 2023). In each of these cases, the spread of information is a critical component to the long-term impacts of that information. To address what makes Twitter posts more or less likely to be successfully spread, we evaluated the statistical properties of more than 15 million posts.

### Methods

The Twitter dataset used in this study was collected by Zhao et al. (2023), who examined political polarization in online discussions surrounding the COVID-19 pandemic. The dataset consists of publicly available tweets posted between February 2020 and January 2021. For the present study, we focus solely on the linguistic features of tweets, independent of any political classification. The tweetsbotornot2 package in R (Kearney, 2018) was used to assess the likelihood of an account being automated, and any account with a bot probability score above .5 was excluded from the dataset. Given that language use plays a critical role in our analyses, we restricted our dataset to English-language tweets. Non-English tweets were identified and removed using Twitter’s metadata language tag, ensuring consistency in linguistic processing and measurement of language features.

For each post, we know whether it was retweeted. To investigate if concrete posts are more likely to be shared, we computed the concreteness ratings for each post using recent large-scale concreteness norms, including more than 40,000 words, each ranked on a Likert 5-point scale by multiple online participants (Brysbaert, Warriner, et al., 2014). In computing the concreteness of a post, we relied on word-level concreteness ratings from Brysbaert, Warriner, et al. (2014). Not all words in our dataset had an assigned concreteness value in this database. When a word lacked a concreteness score, it was excluded from the averaging process rather than being imputed or assigned a default value. Approximately 12.43% of words in the Twitter dataset lacked a rating, leaving our concreteness computations based on the remaining 87.57% of words. This approach ensures that our computed post-level concreteness scores reflect only words with established ratings. For each psycholinguistic feature (e.g., concreteness, age of acquisition), we matched words in each post to established norms and computed the post-level score by averaging the values of all matched words.

We also computed age of acquisition (Kuperman et al., 2012), valence, arousal, dominance (Warriner et al., 2013), and humour (Engelthaler & Hills, 2018) for each post. Age of acquisition refers to the age when people learn a particular item (Elsherif et al., 2023). Concreteness refers to specific and tangible language (Williams & Bizup, 2017). Valence is a characteristic of emotions. For example, “negative” emotions like anger and fear have a negative valence but positive emotions like joy have a positive valence (National Institute of Mental Health (NIMH), 2023).

For age of acquisition, we used word-level ratings from Kuperman et al. (2012) as it was the most comprehensive age of acquisition resource available, and the ratings contribute substantially to the lexical-decision task of the English Lexicon Project (Kuperman et al., 2012). For valence, arousal, and dominance, we used the norm ratings from Warriner et al. (2013), whose ratings correlate with previous affective norm datasets while covering many more words and more commonly used by existing studies. For humour, we used norm ratings from Engelthaler and Hills (2018), which is one of the few well-validated existing norm ratings for humour.

To ensure data quality and consistency, we applied a series of preprocessing steps to the dataset before analysis. First, we removed duplicate entries to retain only unique posts. Next, all text was converted to lowercase to standardise formatting and prevent mismatches due to capitalisation. To eliminate extraneous elements, we removed URLs and hashtags. Additionally, we filtered out numbers and punctuation to focus on meaningful words. Since social media posts often contain emojis and other non-standard symbols, we used Unicode-based regular expressions to exclude such characters. Finally, we removed stopwords (e.g., the, and, is) using a predefined list of English stopwords (R Core Team, 2024) to ensure that non-informative, high-frequency words did not bias the analysis. After these preprocessing steps, the cleaned text was used for subsequent linguistic feature extraction and engagement analysis.

For a post, we took each word and found its value in the associated norms (if the word appeared in the norms) and then calculated the average value of the words in the post. We ran both a single logistic regression model for all variables and a logistic regression for each topic with all variables using whether the post had been retweeted as the dependent variable, with concreteness, age of acquisition, valence, arousal, dominance, and humour as independent variables. We also applied a Bonferroni correction for multiple hypothesis testing. The dataset was categorised by Zhao et al. (2023) into 35 topics using Latent Dirichlet Allocation (LDA) (McCallum, 2002). The 35 topics cover a wide range of issues, including sports, religion, show business, cooking, the 2020 U.S. election, diplomacy, and national security (Zhao et al., 2023). We used the scale() function in R to standardise the scores of each psycholinguistic feature.

### Results

**Figure 1.**
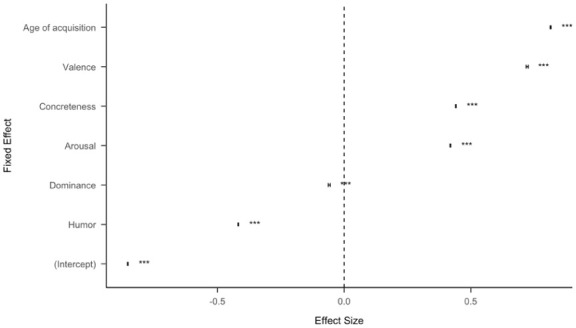
Standardized effect size (coefficient estimates) of psycholinguistic features for all Twitter data (15,565,582 Tweets). The effect size represents the coefficient estimate of a logistic regression model to predict whether a tweet was retweeted. The error bars represent 95% confidence intervals. Some of the confidence intervals are too small to be seen.

For the entire dataset with all topics combined in a regression, all predictors are significant (*p<.001*), with age of acquisition, concreteness, valence, and arousal positively predicting the likelihood of retweet, while humour and dominance negatively predicting the likelihood of retweet (see Figure 1 and Table S1 for the full regression table). This supports the primary claim that concreteness independently influences the appeal of information. In a regression with topics and concreteness as independent variables while “whether a tweet was retweeted” as dependent variable, concreteness remains predictive (β=.014, *SE*=0.00059, *CI*=[0.012, 0.015], *p*<.001), which indicates that it is not more concrete topics driving the main effect of concreteness.

**Figure 2.**
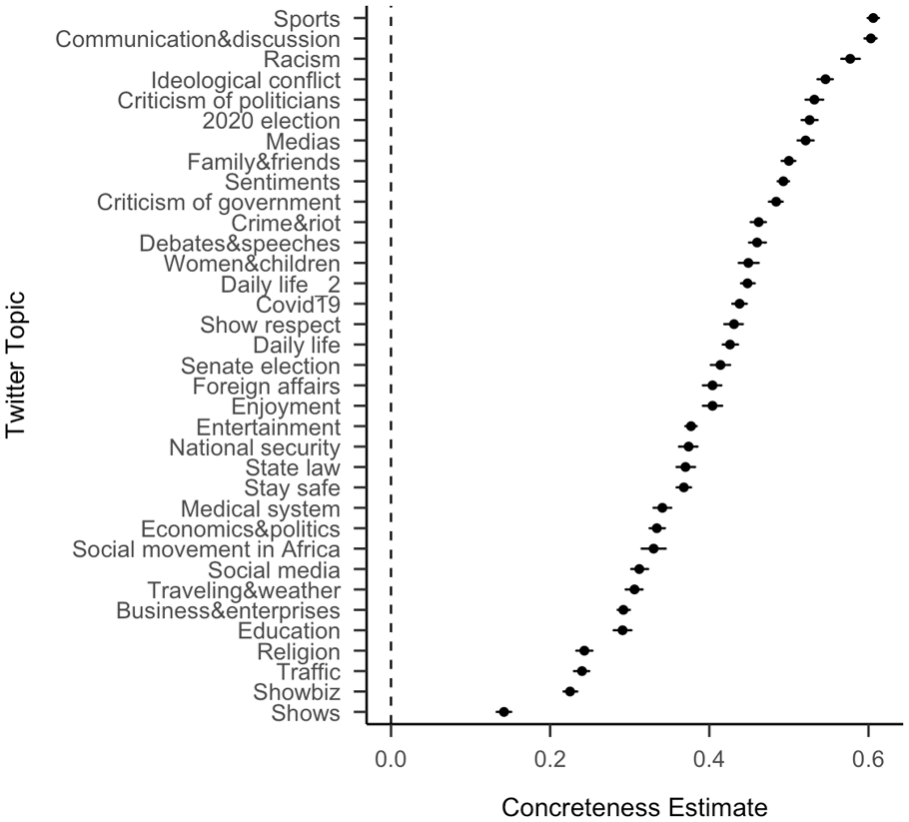
Standardised effect size (coefficient estimates) of concreteness across Twitter topics from separate regression models for data from each topic. The effect size represents the coefficient estimate of a logistic regression model to predict whether a tweet was retweeted. The error bars represent 95% confidence intervals.

Figure 2 shows that within topics, we see the same effect of concreteness, with more concrete posts increasing the likelihood of retweeting. For example, the concreteness effect is particularly strong for the topics of “racism,” “2020 election,” “criticism of politicians” and “ideological conflict,” while less for topics such as “shows,” “traffic,” “showbiz,” and “religion.” The trend for the influence on concreteness in political language may be a general effect, also supported by Bhatia and Walasek’s (2016) discovery that the New York Times used increasingly more concrete language as election dates approached (from 1987 to 2007). In the Supplemental Materials (Table S1 and Figures S1–S5), we show the effects for additional psycholinguistic features within topics. As above, age of acquisition, concreteness, arousal, and valence positively predict whether the tweets have been retweeted across all the topics, while dominance predicts retweeting with differences across topics. For example, less dominant tweets under the topic “enjoyment” tend to be retweeted, and more dominant tweets under the topic “2020 election” tend to be retweeted. Humour negatively predicts retweeting, with more humorous tweets being less likely to be retweeted.

## Study 2

In Study 2, we ask whether we observe similar effects for Reddit posts. Reddit posts represent a different format from Tweets and offer a way to communicate ideas to a target community with respect to a specific topic (or subreddit).

### Methods

Reddit is a social network with elements of news aggregation, content rating, and forums. We scraped Reddit posts using Python. The scraping started on 28 December 2024 and ended on 8 January 2024. We scraped the front page, which includes posts from a range of subreddits. We also scraped posts directly from popular subreddits, including “askreddit,” “explainlikeimfive,” “futurology,” “gaming,” “getmotivated,” “lifeprotips,” “news,” “nottheonion,” “science,” “showerthoughts,” “space,” “sports,” “todayilearned,” “upliftingnews,” “worldnews.” These 15 subreddits do not focus on gifs, music, pictures or videos and are among the top 30 popular subreddit communities (as listed at https://www.reddit.com/best/communities/1/). Overall, we collected 90,347 distinct Reddit posts. Because the frontpage consisted of different subreddits, we removed posts from those subreddits included in the front page that focused on music/gif/video/pictures. These removed subreddits included “aww,” “creepy,” “earthporn,” “food,” “listentothis,” “photoshopbattles,” “pics,” “videos,” “funny,” “gifs,” “announcements,” “blog,” “DIY,” “documentaries,” “mildlyinteresting,” “music,” and “oldschoolcool.” For each post, we captured the text and the number of upvotes it received.

After exclusions, our dataset consisted of 50,517 Reddit posts. Similar to Study 1, we used word-level concreteness ratings from Brysbaert, Warriner, et al. (2014). Words without a concreteness score were excluded from the averaging process to ensure that our computed post-level concreteness scores reflect only words with established ratings. Approximately 11.44% of words in the Reddit dataset lacked a rating, leaving our concreteness computations based on the remaining 88.56% of words. Similar to Study 1, we applied preprocessing steps to the dataset and computed the concreteness, age of acquisition (Kuperman et al., 2012), valence, arousal, dominance (Warriner et al., 2013), and humour (Engelthaler & Hills, 2018) for each Reddit post. Then we ran a multiple linear regression using the number of upvotes as the dependent variable, with concreteness, age of acquisition, valence, arousal, dominance, and humour as the independent variables (Table S2). We also ran regressions for the data from each subreddit. We applied a Bonferroni correction for multiple hypothesis testing. We used standardised effect sizes by using the function *scale* in R for analysis.

### Results

**Figure 3.**
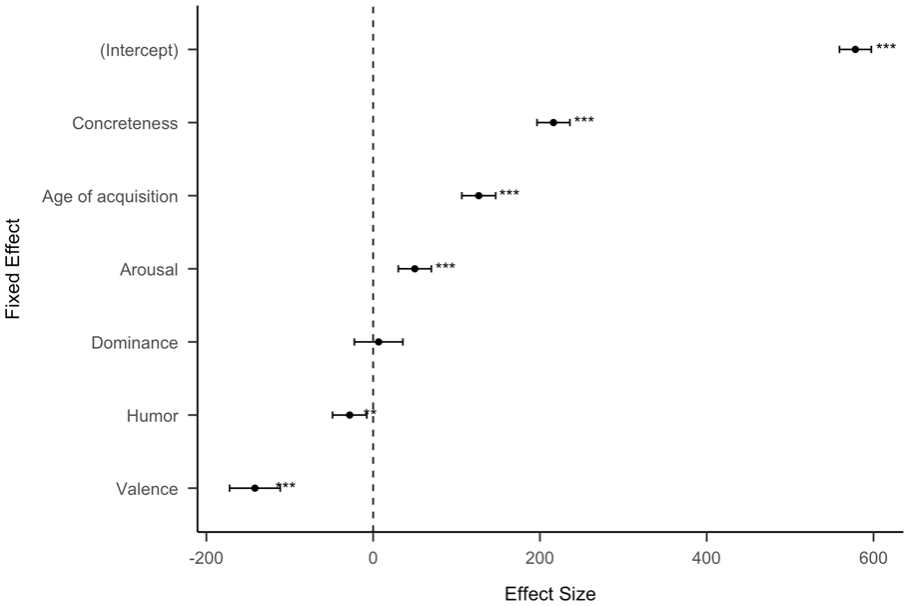
Standardized effect size (coefficient estimates) of psycholinguistic features for all Reddit data (50,517 posts). The effect size represents the coefficient estimate of a multiple linear regression model to predict the number of times each post is upvoted. The error bars represent 95% confidence intervals.

For the entire Reddit dataset with subreddits combined (Figure 3), we found that concreteness positively predicts the number of upvotes, together with age of acquisition, and arousal. Valence and humour negatively predict the number of upvotes, and dominance is not predictive. To be more specific, every unit point increase in the effect size of concreteness is equivalent to 216.23 more upvotes. (Table S2).

**Figure 4.**
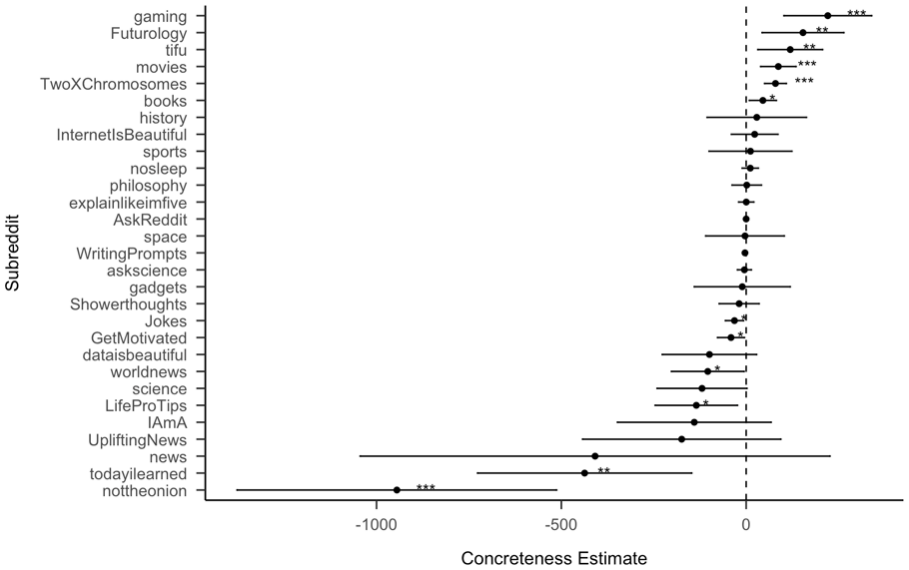
Standardized effect size (coefficient estimates) of concreteness across subreddits from separate regression models for data from each subreddit. The effect size represents the coefficient estimate of a linear regression model to predict the number of times each post is upvoted.

Within subreddits, we found that concreteness of content positively predicts the number of upvotes in 6 of 29 subreddits: “gaming,” “Futurology,” “movies,” “books,” “tifu,” and “twoxchromosomes,” and concreteness of content negatively predicts the number of upvotes in 6 of 29 subreddits: “nottheonion” and “todayilearned,” “LifeProTips,” “worldnews,” “Jokes,” “GetMotivated.” For other subreddits, concreteness was non-significant as a predictor. In the case of the “nottheonion” subreddit, this may indicate that for news perceived as unrealistic or untruthful, greater concreteness in language reduces the likelihood of upvotes. In the Supplemental Material, we provide further information about the other psycholinguistic features (Figures S6–S10). As seen in Figure 4, there is notable variation in the direction of the concreteness effect across different topics—an approximately equal number of topics show a positive or negative relationship between concreteness and upvotes. This suggests that the overall positive main effect of concreteness on Reddit is moderated by topic. Some topics benefit more from concreteness than others, and the topics that do benefit are also more popular, because although we tried to scrape the same number of posts for each subreddit, some subreddits have a smaller number of distinct posts, and those subreddits with fewer posts tend to show negative effects of concreteness. Therefore, it is difficult to disentangle whether popularity is driving the concreteness effect, or vice versa. This motivates the experiment we present below.

## Study 3

The above two studies indicate that the likelihood of sharing or upvoting Twitter and Reddit posts is positively correlated with their concreteness. However, as the other variables indicate, this could be for a variety of reasons, such as content alignment with other news sources and complex interactions between different variables. A causal demonstration of concreteness requires an experimental manipulation. Additionally, because averaging concreteness scores may obscure meaningful within-post variation, we need a more controlled approach to manipulate concreteness. In order to achieve this, we ran a controlled experiment that independently manipulated concreteness in an ecologically valid context using social media posts. We used ChatGPT to slightly alter posts to make two new posts that were more and less concrete. Participants then indicated which post they would be more likely to share. This directly tests the impact of concreteness and allows us to additionally control for other features of language variation as described below.

### Methods

The experiment and analysis were pre-registered (https://osf.io/qp8ms). We received ethics approval from the University of Warwick HSSREC. The experiment has a within-subjects design. Each participant was shown a sequence of 55 questions in a random order. In each question, participants were presented with a pair of posts that slightly differed in their language. They were asked to select the one they are more likely to upvote(like). Participants were presented with the prompt “Among the following two Reddit posts, please select the one you are more inclined to upvote or share.” A one-sample *t*-test with the proportion of more-concrete options chosen was used to test for a preference for concreteness. We also fit a logistic regression with the choice of the more-concrete option as the dependent variable and the differences in psycholinguistic features between pairs of statements as independent variables.

For example, one pair of posts were “Twitter profiles displaying explicit gender pronouns (such as she/her, he/him, and they/them) have seen a marked rise in prevalence since the last decade” (more concrete) and “Twitter user bios denoting personal pronouns have become notably widespread in the past decade” (less concrete).

For the stimuli selection procedure, we began by collecting posts from Reddit’s front page on 23 December, forming the basis of our dataset. We filtered out posts from subreddits focused on visual, auditory, or animated content, ensuring our stimuli were text-based. Posts were then ranked by concreteness, with the median five posts from each relevant category selected. Further exclusions were made for excessively long, irrelevant, or unpopular posts. The selected posts were then edited by ChatGPT to create more and less concrete versions, followed by manual quality checks. Length and other linguistic features were controlled between pairs of statements. Finally, we validated the effectiveness of these manipulations by using concreteness ratings from Brysbaert, Warriner, et al. (2014) to compute averaged concreteness score for each post across words, ensuring that the manipulated content differs on concreteness (see the more detailed stimuli selection procedure in the pre-registration: https://osf.io/qp8ms).

Two attention checks were embedded within the questions, and participants who failed either check were excluded from the analysis. For example, in one of the attention checks, the statements were: “The Pentagon is using smart technology to make tough choices about weapons that can act on their own. Many experts believe the U.S. will have fully self-operating lethal weapons in a few years. [This is an attention check. These two options are the same. Please choose the other option].” and “The Pentagon is using smart technology to make tough choices about weapons that can act on their own. Many experts believe the U.S. will have fully self-operating lethal weapons in a few years.”

The study was conducted online using the Qualtrics online survey platform and Research Experience platform of the University of Warwick. 192 students consented to taking part in the study. Twenty-three participants failed the attention checks. Two participants did not finish the study. One hundred and sixty-seven participants remained in the dataset.

### Results

A one-sample *t*-test showed that participants chose the more-concrete option significantly more often than the less-concrete option (*t*(166) = 15.36, *p*<.001). A non-parametric Wilcoxon signed-rank test supported this result (*V=*13249, *p*<.001).

**Figure 5.**
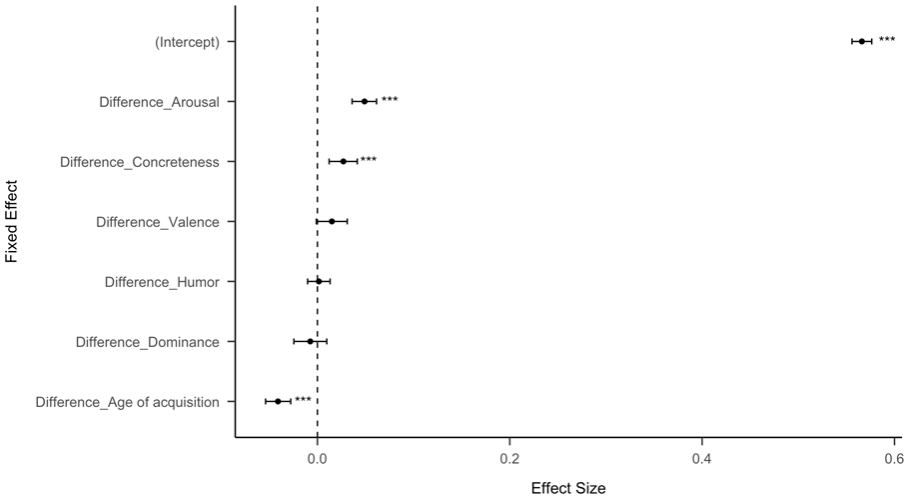
Standardized effect size (coefficient estimates) of psycholinguistic features for experiment data (55 pairs of posts). The effect size represents the coefficient estimate of a logistic regression model to predict whether a more concrete option was chosen. The error bars represent 95% confidence intervals.

**Figure 6.**
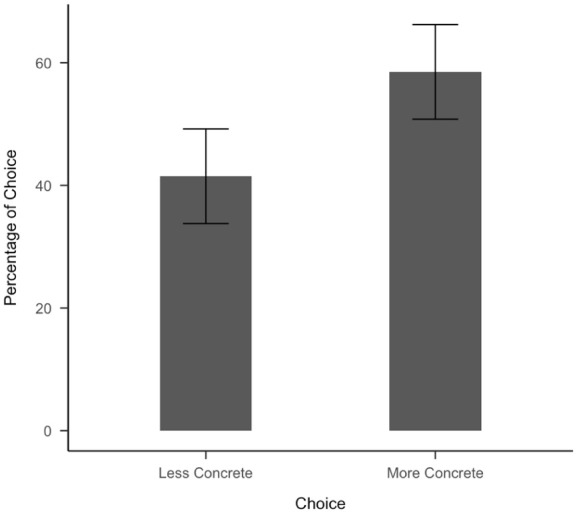
Percentage of choice for experiment data (55 pairs of posts).

**Figure 7.**
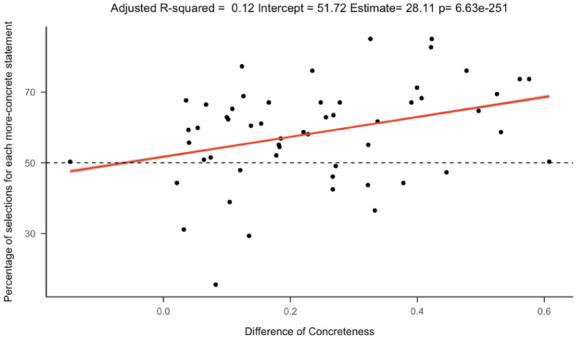
Percentage of selections for each more-concrete statement against the difference of concreteness between each pair. The points represent each pair of posts, with the difference in concreteness between the posts (*x*-axis) and the number of participants who chose the more concrete post. The red line shows a linear regression fit.

A multiple logistic regression found that more concrete posts were more likely to be chosen, with a strong effect size for the intercept in the regression. In addition, differences in concreteness, age of acquisition, arousal, all significantly predicted the likelihood of choosing the more concrete option. This indicates that higher concreteness and arousal increase the likelihood of participants choosing the more-concrete option while lower age of acquisition increases the likelihood of participants choosing the more-concrete option (Figures 5 and 6). We also ran a linear regression with the percentage of selections for each more-concrete statement as the dependent variable and the difference of concreteness between each pair as the independent variable and found that the greater the difference of concreteness between each pair of post, the more likely participants would choose the more-concrete option (Figure 7). The intercept confidence interval spans 50%, which aligns with there being no preference when posts have the same concreteness (Table S3).

## Discussion

Concreteness is a property of language that shapes the way we communicate and the way language changes over time (Brysbaert, Warriner, et al., 2014; Hills & Adelman, 2015; Hills et al, 2017; Li et al., 2024). Here, we examined if this effect would be observable on the likelihood of sharing social media posts. We used data from two different social media platforms to examine the connection between concreteness and the behaviours of retweeting and upvoting. In Study 1, we examined if people tend to retweet more concrete Twitter posts using around 15 million Twitter posts consisting of 35 different topics. In Study 2, we analysed if concrete information is more likely to be upvoted using around 50 thousand scraped posts from Reddit. Both studies converged on the same conclusion: concreteness positively predicts information sharing/upvoting. In Study 3, we verified these findings with a behavioural experiment that showed that participants prefer more concrete statements.

Overall, we found compelling evidence that concrete language is preferred by people when deciding between pieces of information. People are more likely to share, or propagate, concrete information. This is consistent with the observation that concrete language is more likely to survive story retellings in the laboratory and to persist in language over hundreds of years (Li et al., 2024). With the current study providing supporting evidence in the form of preferences for sharing more concrete information in modern media environments, people’s preferences for sharing concrete language are likely to be an important factor in the rise of concrete language more generally (see Hills & Adelman, 2015).

While our findings highlight the role of linguistic concreteness in shaping engagement on social media, it is important to acknowledge that engagement metrics (e.g., retweets, upvotes) are influenced by additional extrinsic factors that were not explicitly modelled in our analyses. For instance, the follower count of the original poster can significantly affect the visibility and spread of a post, as accounts with larger audiences are more likely to have their content amplified. Moreover, platform-specific algorithms play a critical role in content dissemination by prioritising certain types of posts based on factors such as recency, interaction history, or controversiality (Lorenz-Spreen et al., 2021). These algorithmic interventions may introduce biases that are independent of a post’s linguistic features. Although these factors were not controlled for in our models—primarily due to data availability constraints—it is important to consider whether they systematically interact with linguistic concreteness. If, for example, platform algorithms or high-profile users tend to favour concrete language, then our observed effects may be partially mediated by these extrinsic influences. Conversely, if algorithms prioritise more abstract or provocative content, our results might be an underestimate of the true effect of concreteness on engagement. These concerns are partly allayed by Study 3, which also found a preference for greater concreteness, despite the absence of such extrinsic factors.

Also, while our study highlights the role of concreteness in shaping engagement in online communication, our results also show that other linguistic features, such as arousal and valence, sometimes have stronger effects on engagement metrics. This suggests that while concreteness is an important driver of information propagation, it operates within a broader landscape of word-level properties that shape online discourse. From the perspective of information markets, this finding is consistent with prior research showing that emotionally charged content—particularly highly arousing or positively/negatively valenced language—can enhance engagement by capturing attention and eliciting stronger reactions (Berger & Milkman, 2012; Ito et al., 1998; Robertson et al., 2023). In fast-paced digital environments, where users are constantly exposed to competing information, high-arousal content may spread more rapidly simply because it is more attention-grabbing, whereas concreteness may play a stronger role in influencing credibility and comprehension.

Furthermore, while our study relies on Brysbaert, Stevens, et al.’s (2014) concreteness norms, these ratings are based on isolated words and static judgments, which may not fully capture how concreteness functions in dynamic, context-dependent online discourse. Words can shift in meaning depending on phrasing and usage (e.g., “cell” as a biological unit vs. a terrorist group), and multi-word expressions are not accounted for in single-word ratings. Additionally, the norms lack coverage for internet slang, technical jargon, and emerging words, leading to missing data that could bias engagement estimates. Future work could use context-based word embeddings to generate concreteness scores dynamically.

One of the limitations of our experiment is that we did not disentangle concreteness and specificity, mainly because we wanted to control the meaning of the statements being rated. The correlation between specificity and concreteness among the terms we use is .255. Future research could treat concreteness and specificity as two different dimensions to investigate their effects and interactions.

Despite these limitations, the robustness of our findings across platforms suggests that linguistic concreteness remains a meaningful predictor of engagement. Future studies could incorporate more nuanced, dynamic measures of concreteness and examine how it interacts with other linguistic and extrinsic engagement factors, such as author influence, content novelty, and platform algorithms.

Why is concrete language preferred? One potential explanation stems from dual-coding theory (Paivio et al., 1994), according to which memory of abstract content involves only verbal processing, whereas memory of concrete content involves both imaginal and verbal processing. The greater involvement of cognitive processing may make this information more stimulating or satisfying, and people may infer the impact of this on others when sharing information. Further work is needed to connect the preference for concreteness with underlying neural and psychological mechanisms.

Beyond its role in enhancing engagement and retention, concreteness may also interact with other cognitive and emotional mechanisms that influence the spread of fake news. One promising direction for future research lies in understanding how linguistic concreteness interacts with word-level features such as age of acquisition and emotional tone. Future work could examine how concreteness, in combination with humour and early-acquired language, contributes not only to the spread of information but also to the formation of beliefs—whether accurate or not.

Overall, our findings speak to the importance of word features, and specifically concreteness, in online communication. This fits into a broader literature on the role of concreteness in facilitating cognitive processing, in both recognitions, encoding, and later recall. The attributes are critical to the life cycle of information (Hills, 2019), and the present study suggests that we can see that influence even in short-form media. In addition to the rise in concreteness in language more generally (Hills & Adelman, 2015), short-form media is also on the rise (Pilgrim et al., 2024). Future work could look across content forms to evaluate whether or not short-form media is generally higher in concreteness than long-form media, and may in part be facilitating its rise. If the history of communication is partly about communicating more information more rapidly into the heads of others, then concreteness and the properties it carries are deserving of more study and may be critical to understanding how to deliver better content, why some content is stickier than others (e.g., Giese et al., 2020; Jagiello & Hills, 2018).

## Supplemental Material

sj-docx-1-qjp-10.1177_17470218251392831 – Supplemental material for Concrete language enhances sharing of social media posts on Twitter, Reddit, and experimentally Supplemental material, sj-docx-1-qjp-10.1177_17470218251392831 for Concrete language enhances sharing of social media posts on Twitter, Reddit, and experimentally by Danyang Hu, Charlie Pilgrim, Weize Zhao and Thomas T. Hills in Quarterly Journal of Experimental Psychology
