# Supplementary material for: Concrete language enhances sharing of social media posts on Twitter, Reddit, and experimentally
Source: Q J Exp Psychol (Hove). 2025 Oct 21;79(5):1083–93. doi: 10.1177/17470218251392831 (PMC13062457; doi:10.1177/17470218251392831)
Supplement: sj-docx-1-qjp-10.1177_17470218251392831 – Supplemental material for Concrete language enhances sharing of social media posts on Twitter, Reddit, and experimentally [file sj-docx-1-qjp-10.1177_17470218251392831.docx]

**Supplementary Materials for:**

**Concrete language enhances sharing of social media posts on Twitter, Reddit, and experimentally**

Danyang Hu^1*^, Charlie Pilgrim^2^, Weize Zhao^1^, Thomas T. Hills^1^

^1^Department of Psychology, University of Warwick, United Kingdom

^2^Department of Mathematics, University of Leeds, United Kingdom

# Author Note

We have no conflicts of interest to disclose. Correspondence concerning this article should be addressed to Danyang Hu, Department of Psychology, University of Warwick, United Kingdom. Email: [danyang.hu@warwick.ac.uk](mailto:danyang.hu@warwick.ac.uk).

**Figure 1**

*Standardized effect size (coefficient estimates) of age of acquisition across Twitter topics from separate regression models for data from each topic. The effect size represents the coefficient estimate of a logistic regression model to predict whether a tweet was retweeted. Significance levels are denoted as follows: *p < 0.05, **p < 0.01, ***p < 0.001.*


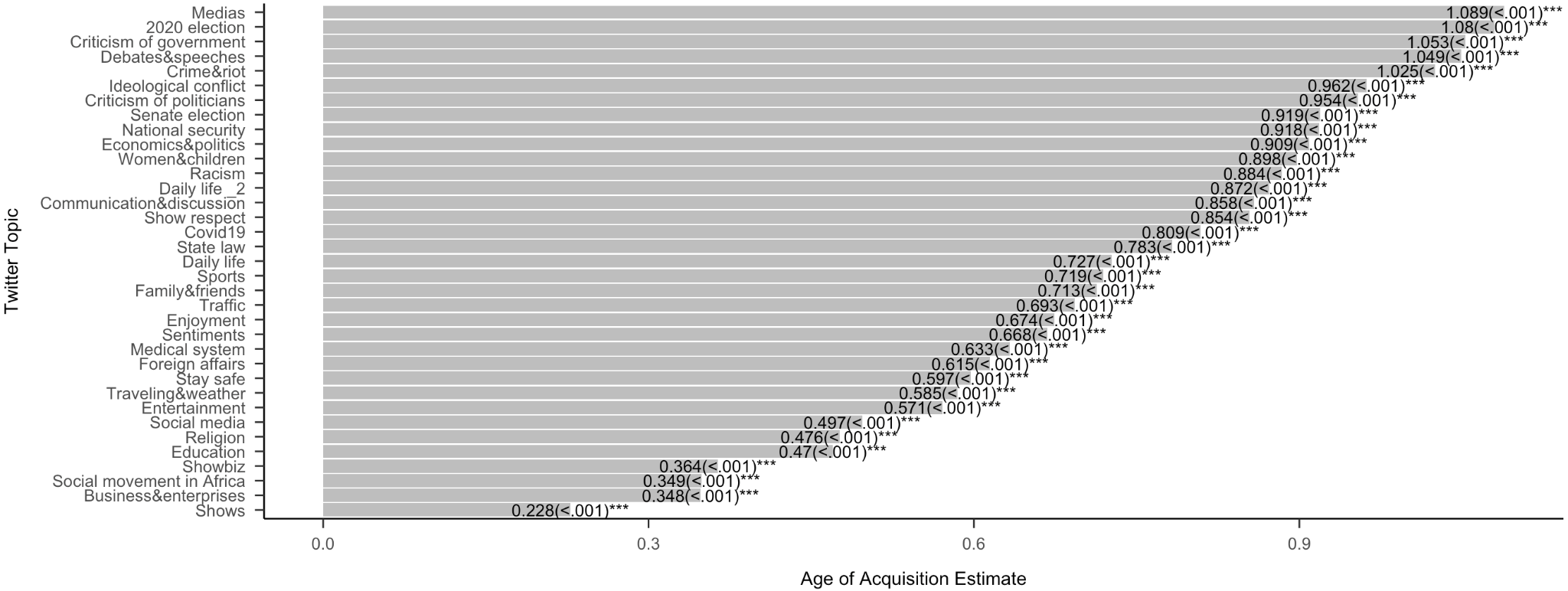


**Figure 2**

*Standardized effect size (coefficient estimates) of arousal across Twitter topics from separate regression models for data from each topic. The effect size represents the coefficient estimate of a logistic regression model to predict whether a tweet was retweeted. Significance levels are denoted as follows: *p < 0.05, **p < 0.01, ***p < 0.001.*

#
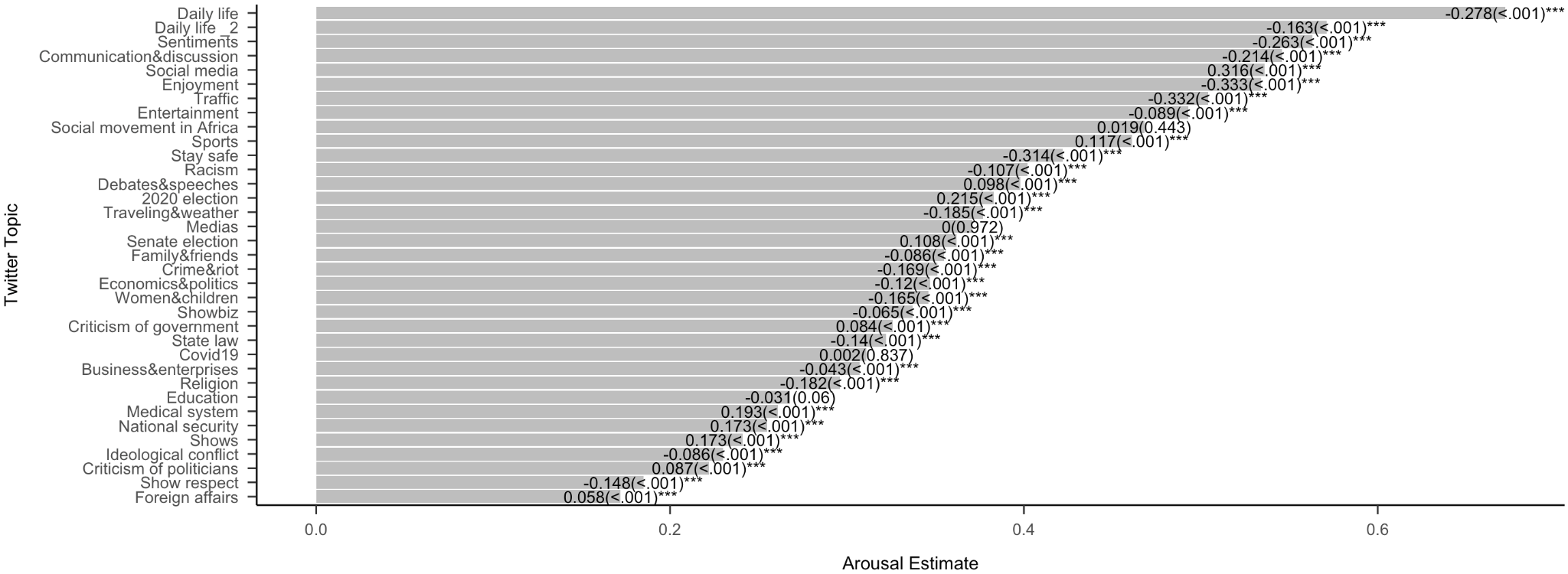


**Figure 3**

*Standardized effect size (coefficient estimates) of dominance across Twitter topics from separate regression models for data from each topic. The effect size represents the coefficient estimate of a logistic regression model to predict whether a tweet was retweeted. Significance levels are denoted as follows: *p < 0.05, **p < 0.01, ***p < 0.001.*


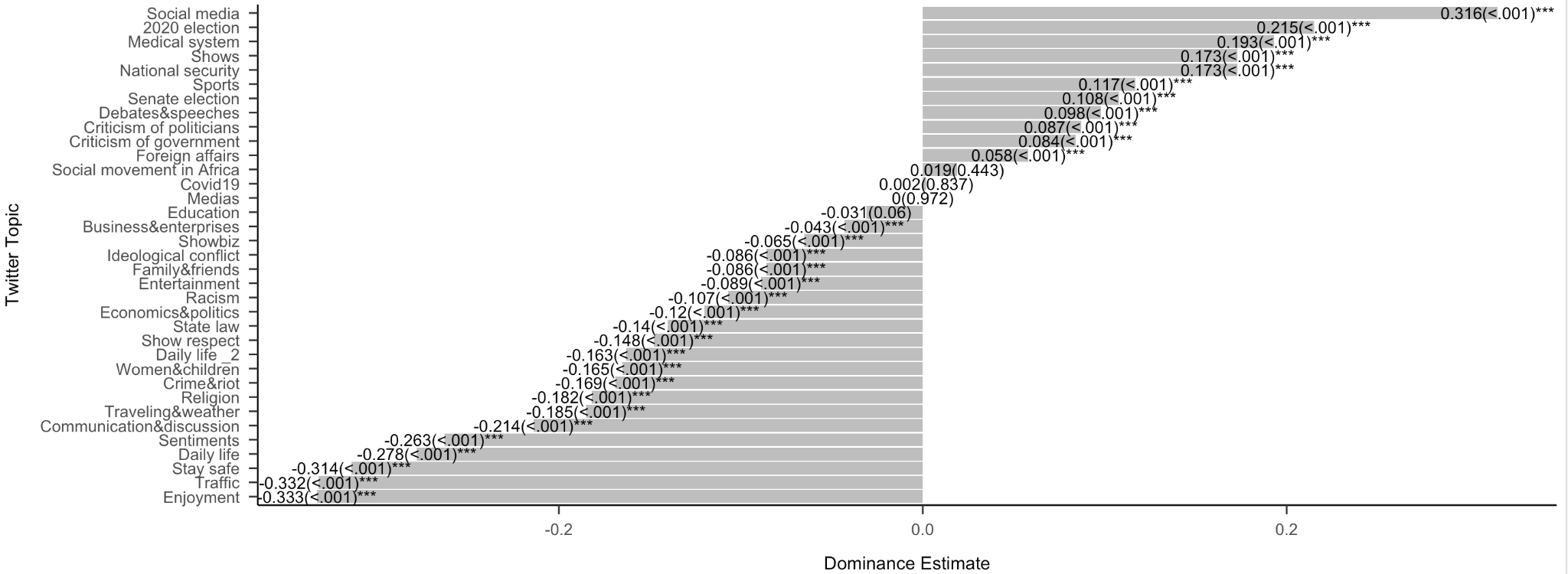


**Figure 4**

*Standardized effect size (coefficient estimates) of humor across Twitter topics from separate regression models for data from each topic. The effect size represents the coefficient estimate of a logistic regression model to predict whether a tweet was retweeted. Significance levels are denoted as follows: *p < 0.05, **p < 0.01, ***p < 0.001.*


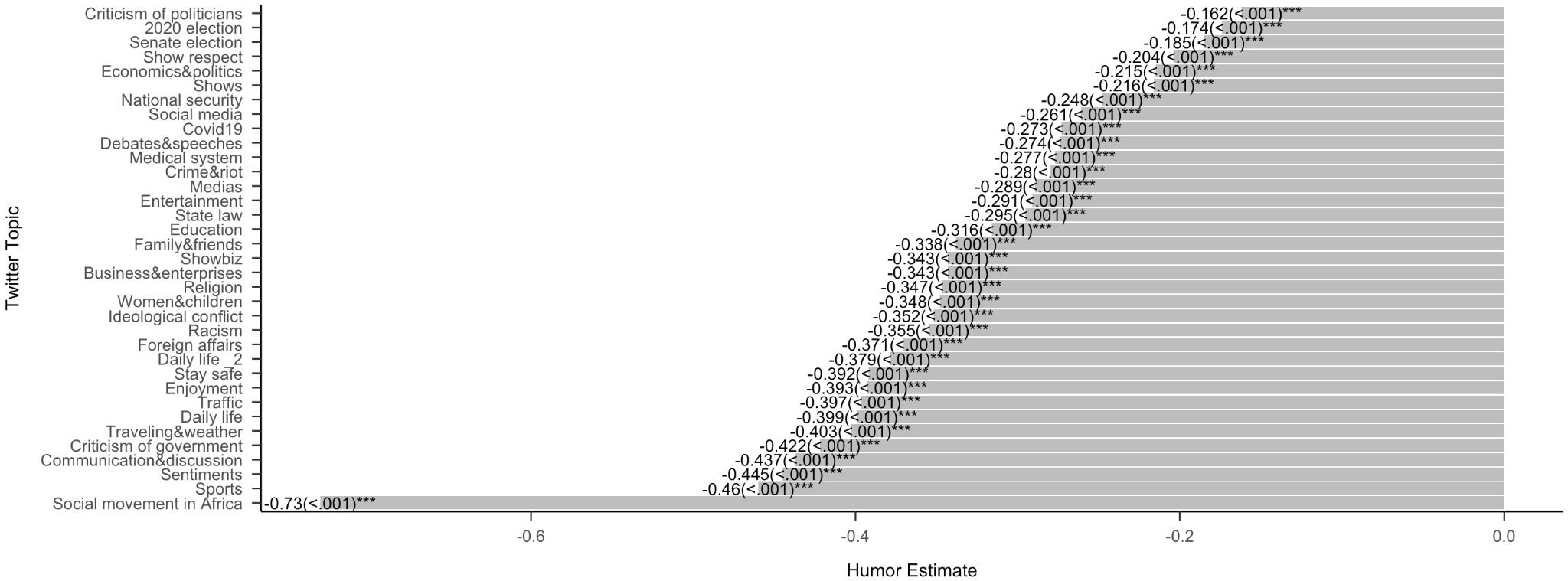


**Figure 5**

*Standardized effect size (coefficient estimates) of valence across Twitter topics from separate regression models for data from each topic. The effect size represents the coefficient estimate of a logistic regression model to predict whether a tweet was retweeted. Significance levels are denoted as follows: *p < 0.05, **p < 0.01, ***p < 0.001.*


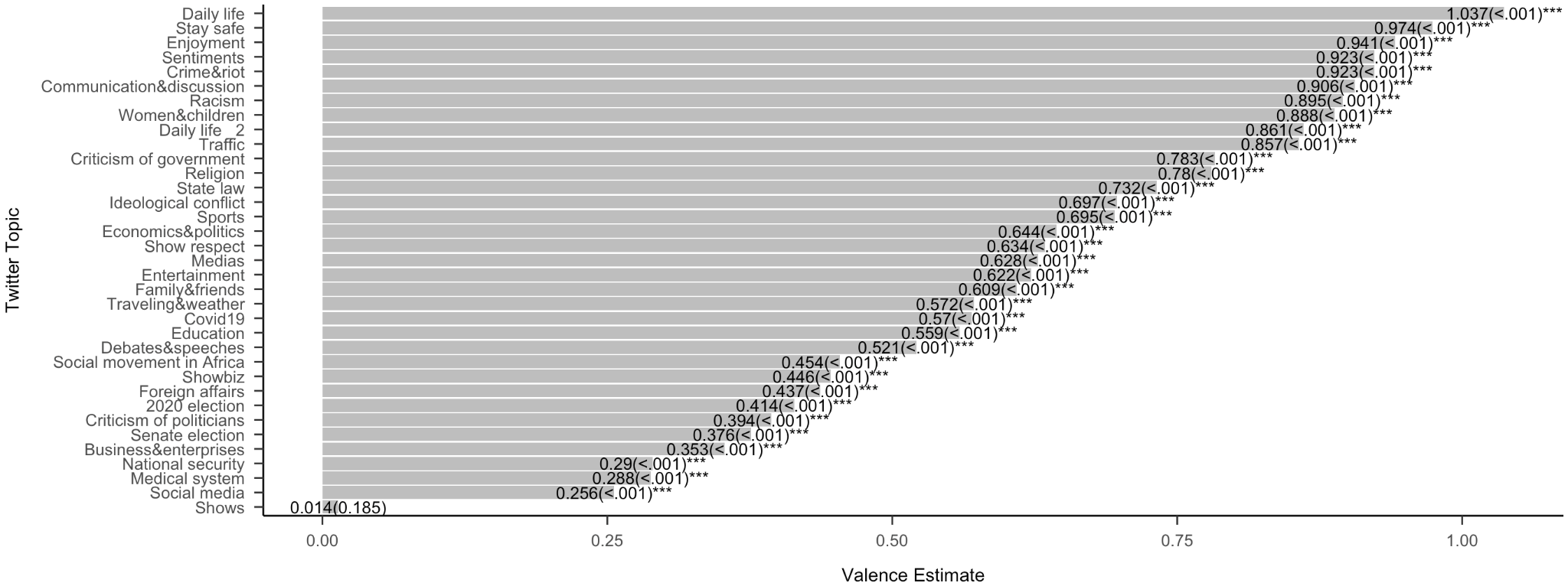


**Figure 6**

*Standardized effect size (coefficient estimates) of age of acquisition across subreddits from separate regression models for data from each subreddit. The effect size represents the coefficient estimate of a linear regression model to predict the number of times each post is upvoted. Significance levels are denoted as follows: *p < 0.05, **p < 0.01, ***p < 0.001.*


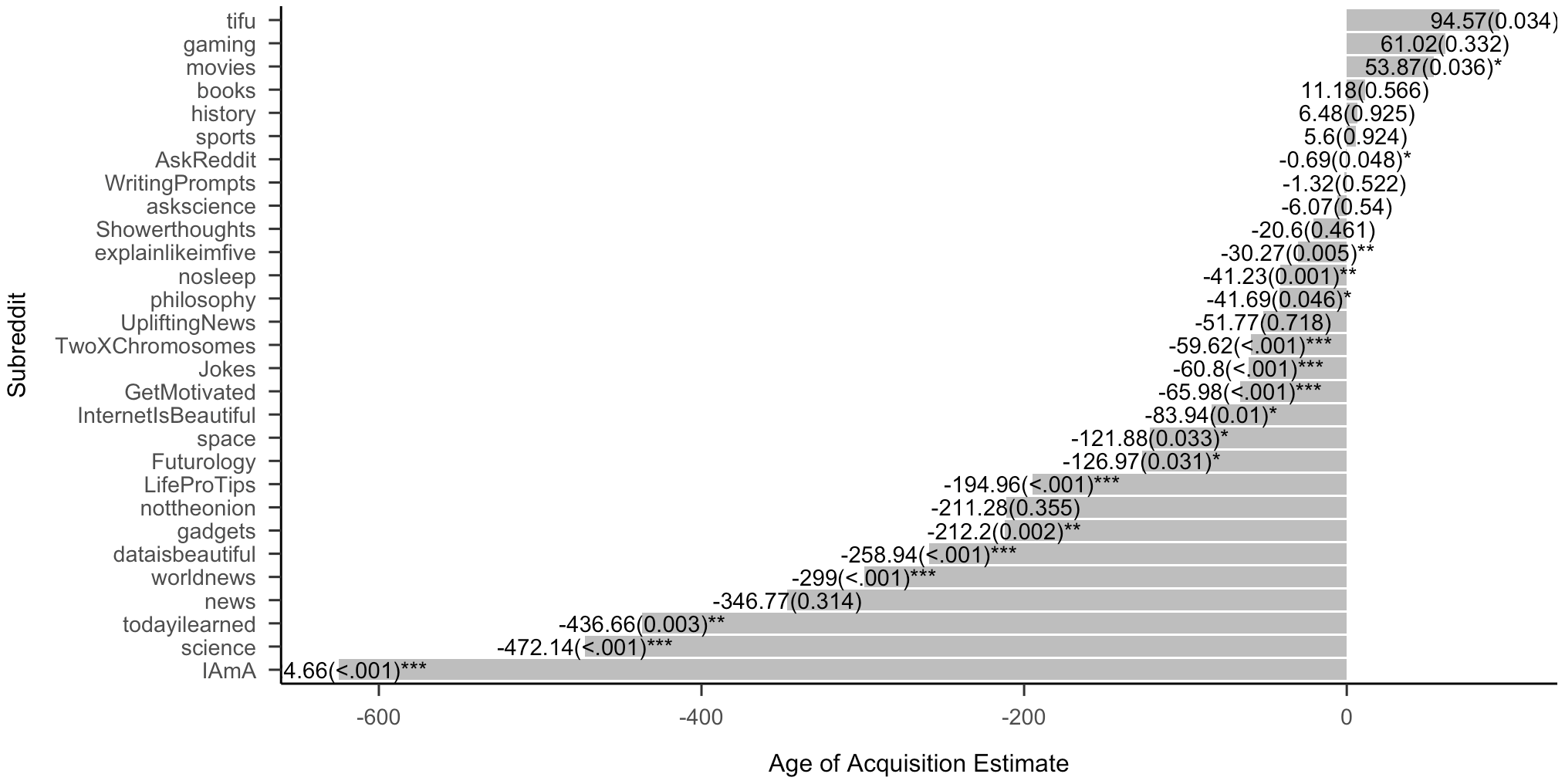


**Figure 7**

*Standardized effect size (coefficient estimates) of arousal across subreddits from separate regression models for data from each subreddit. The effect size represents the coefficient estimate of a linear regression model to predict the number of times each post is upvoted. Significance levels are denoted as follows: *p < 0.05, **p < 0.01, ***p < 0.001.*


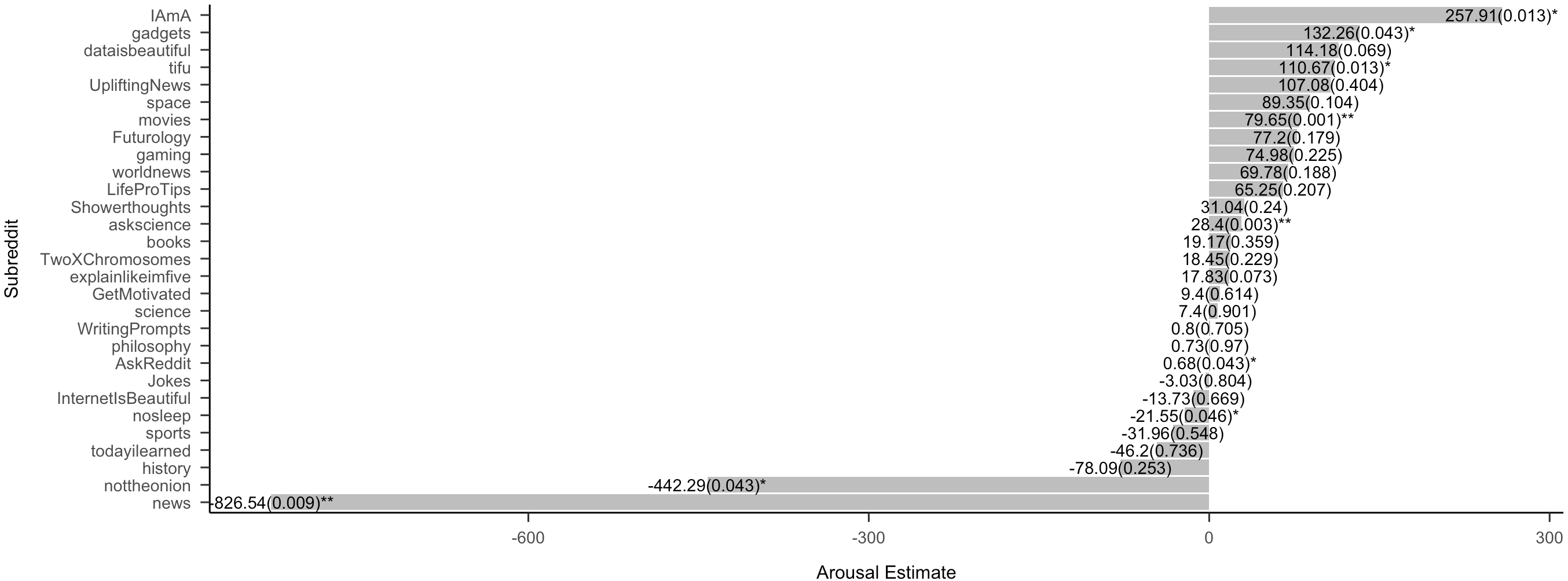


**Figure 8**

*Standardized effect size (coefficient estimates) of dominance across subreddits from separate regression models for data from each subreddit. The effect size represents the coefficient estimate of a linear regression model to predict the number of times each post is upvoted. Significance levels are denoted as follows: *p < 0.05, **p < 0.01, ***p < 0.001.*


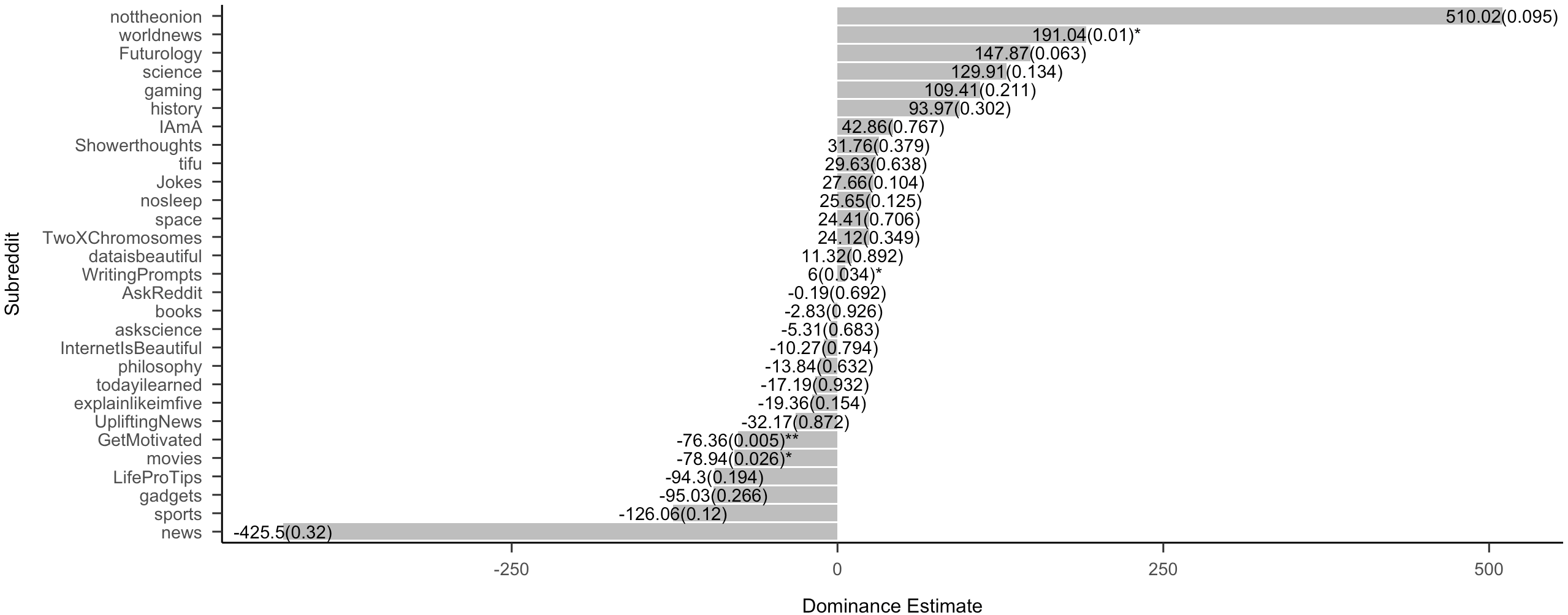


**Figure 9**

*Standardized effect size (coefficient estimates) of humor across subreddits from separate regression models for data from each subreddit. The effect size represents the coefficient estimate of a linear regression model to predict the number of times each post is upvoted. Significance levels are denoted as follows: *p < 0.05, **p < 0.01, ***p < 0.001.*


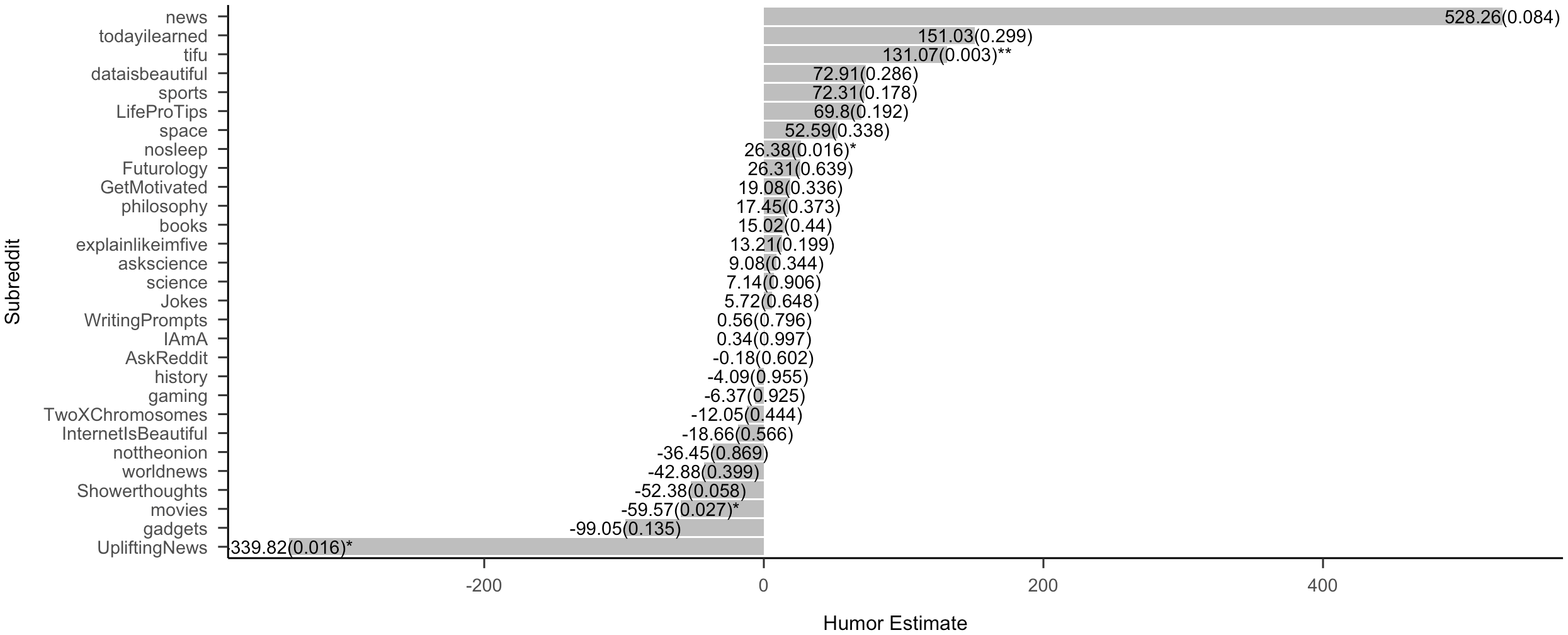


**Figure 10**

*Standardized effect size (coefficient estimates) of valence across subreddits from separate regression models for data from each subreddit. The effect size represents the coefficient estimate of a linear regression model to predict the number of times each post is upvoted. Significance levels are denoted as follows: *p < 0.05, **p < 0.01, ***p < 0.001.*


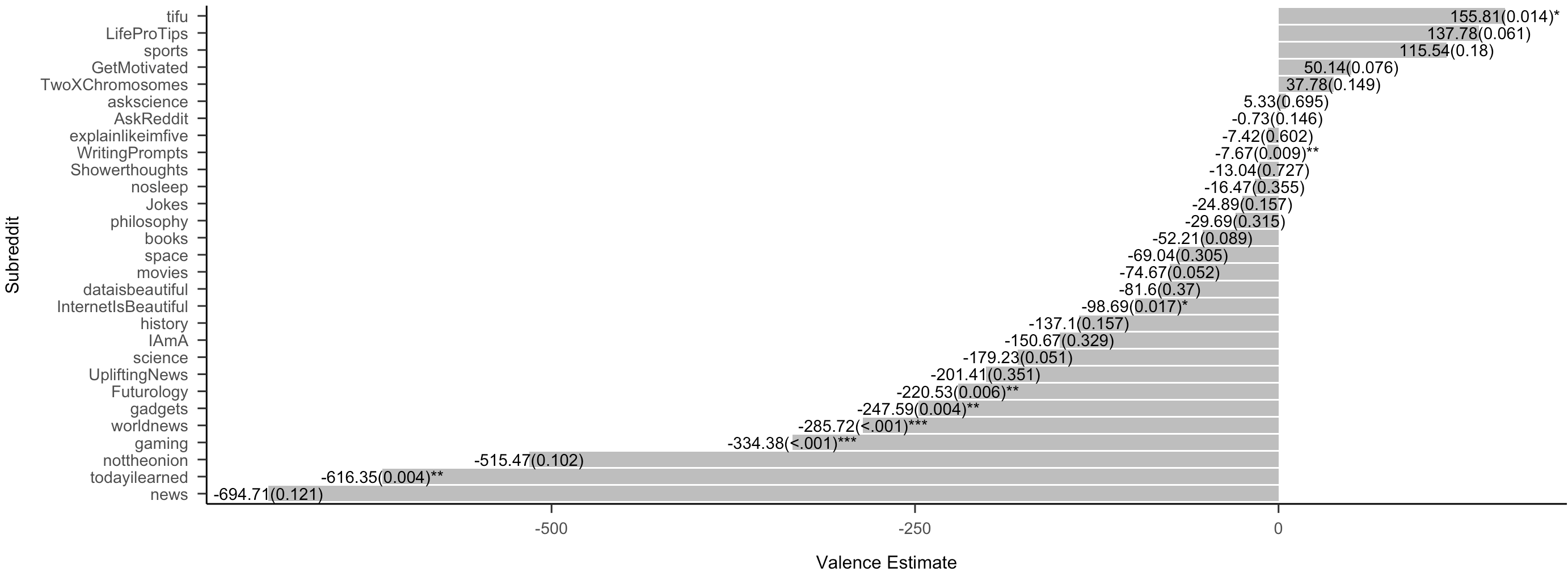


**Table 1**

*Standardized effect sizes of linguistic features predicting Twitter retweets for all topics combined. Results of a logistic regression model to predict whether a tweet was retweeted for the entire dataset, showing coefficient estimates and significance levels for each independent variable. Significance levels are denoted as follows: *p < 0.05, **p < 0.01, ***p < 0.001.*

| Fixed effects | Effect size | 95% CI | P |
| --- | --- | --- | --- |
| (Intercept) | -0.85*** | 0.855 to 0.853 | <.001 |
| Age of acquisition | 0.81*** | 0.813 to 0.817 | <.001 |
| Valence | 0.72*** | 0.718 to 0.727 | <.001 |
| Arousal | 0.42*** | 0.418 to 0.421 | <.001 |
| Concreteness | 0.44*** | 0.439 to 0.442 | <.001 |
| Humor | -0.42*** | -0.419 to -0.416 | <.001 |
| Dominance | -0.059*** | -0.0626 to -0.0547 | <.001 |

**Table 2**

*Standardized estimates of linguistic features for all Reddit posts. Results of a regression model to predict the number of upvotes for the entire dataset, showing coefficient estimates and significance levels for each independent variable. Significance levels are denoted as follows: *p < 0.05, **p < 0.01, ***p < 0.001.*

| Fixed effects | Effect size | 95% CI | P |
| --- | --- | --- | --- |
| (Intercept) | 578.27*** | 559.15 to 597.39 | <.001 |
| Age of acquisition | 126.62*** | 106.34 to 146.91 | <.001 |
| Valence | -141.75*** | -172.10 to -111.40 | <.001 |
| Arousal | 49.96*** | 30.15 to 69.77 | <.001 |
| Concreteness | 216.23*** | 196.52 to 235.93 | <.001 |
| Humor | -28.19** | -48.72 to -7.66 | 0.007 |
| Dominance | 6.53 | -22.55 to 35.62 | 0.66 |

**Table 3**

*Standardized effect sizes of linguistic features predicting choosing the more concrete option. Results of a logistic regression model to predict whether a more concrete post was chosen to be shared, showing coefficient estimates and significance levels for each independent variable. Significance levels are denoted as follows: *p < 0.05, **p < 0.01, ***p < 0.001.*

| Fixed effects | Effect size | 95% CI | P |
| --- | --- | --- | --- |
| (Intercept) | 0.57*** | 0.56 to 0.58 | <.001 |
| Diff_Age of acquisition | -0.041*** | -0.054 to -0.028 | <.001 |
| Diff_Valence | 0.015 | -0.0011 to 0.031 | 0.069 |
| Diff_Arousal | 0.049*** | 0.036 to 0.061 | <.001 |
| Diff_Concreteness | 0.027*** | 0.012 to 0.041 | <.001 |
| Diff_Humor | 0.0015 | -0.010 to 0.013 | 0.80 |
| Diff_Dominance | -0.0074 | -0.024 to 0.0097 | 0.40 |

**Table 4**

*Standardized effect sizes of linguistic features and length predicting choosing the more concrete option. Results of a logistic regression model to predict whether a more concrete post was chosen to be shared, showing coefficient estimates and significance levels for each independent variable. Significance levels are denoted as follows: *p < 0.05, **p < 0.01, ***p < 0.001.*

| Fixed effects | Effect size | 95% CI | P |
| --- | --- | --- | --- |
| (Intercept) | 0.58*** | 0.57 – 0.59 | <.001 |
| Diff_Age of acquisition | -0.059*** | -0.072 – -0.045 | <.001 |
| Diff_Valence | 0.0032 | -0.013 – 0.019 | 0.69 |
| Diff_Arousal | 0.037*** | 0.025 – 0.049 | <.001 |
| Diff_Concreteness | 0.018** | 0.004 – 0.032 | 0.0095 |
| Diff_Humor | 0.0047 | -0.007 – 0.016 | 0.43 |
| Diff_Dominance | 0.0089 | -0.008 – 0.026 | 0.31 |
| Diff_Length | -0.045 | -0.057 – -0.033 | <.001 |
